# Authors’ reply

**DOI:** 10.1192/bjb.2020.41

**Published:** 2020-06

**Authors:** David Rigby, Lynsey McAlpine

**Affiliations:** email: david.rigby@nhs.net

We are delighted that the publication of our article has generated a debate around the Section 12 approval process and welcome the opportunity to respond to the three letters. Before responding to some of the criticisms of our article, we think that readers would benefit on some narrative on why we chose to publish this article. At a similar time to attending a Section 12 approval course, the first author had also attended an advanced life support (ALS) course. These courses have some similarities in that they are 2-day events with teaching on performing specialised tasks which are required in order to work in certain specialties or positions of seniority after accreditation from a respected body.

However, the author's experience of the two courses also had notable differences. To name a few: the ALS course came with an extensive manual, knowledge of which was tested in a multiple choice question; the large majority of the course was spent undertaking simulations of the tasks in which the course was accrediting competence; and IDs were checked and significantly late arrivals would have resulted in course failure, and therefore the course was promptly attended.

By contrast, the Section 12 course had several late arrivals and some early leavers. There were no ID checks. Teaching, although of a high standard, was mostly lecture based and didactic and, most worryingly, a significant minority of attendees spent large amounts of time using their phones during the course. There was no simulation training on performing Mental Health Act assessments (MHAAs) in the course.

Although anecdotal, these differences should cause concern to those with responsibility for Section 12 approval. As we demonstrated in our article, our belief that the difference in engagement in these two courses is due to lack of assessment in the Section 12 approval process is based on pedagogical research that shows that students will not engage in effective learning when they are not assessed.

We accept Dr Khwaja's criticism that we do not have contemporaneous evidence of a lack of knowledge or skills in Section 12-approved doctors but argue that – in contrast to the current situation – the onus should be on Section 12 approval courses to establish through assessment that participants have in fact acquired appropriate knowledge and skills.

We note Dr Khwaja's suggestion to change the title of our article, but we stand by our original title as we believe that there are concerns in multiple domains of the Section 12 approval process. For instance, many National Health Service trusts only allow Section 12-approved doctors to perform MHAAs. The MRCPsych exams do not assess knowledge of mental health law. Therefore, it is entirely possible for a doctor to receive Section 12 approval who has never performed an MHA, simulated or real, or who did not attend the approval course, instead asking a colleague to sign in, or attended the course but chose to engage with their phone rather than the course content. Essentially, UK doctors receiving Section 12 approval will not have had any formal assessment in their knowledge and skills in relation to performing an MHAA.

Dr Khwaja notes that international candidates are required to have evidence of having undertaken supervised MHA assessments. Surely this disparity should be corrected and extended to all Section 12 approvals in the UK? He also notes that only a basic working knowledge of the MHA is required to conduct an assessment, but this should not negate responsibility for assessing Section 12 doctors for any knowledge of the MHA.

The authors are aware that Section 12 approval is not required to take part in an MHA but argue that this is even more reason to ensure that Section 12 doctors have the appropriate knowledge and skills to undertake assessments if they are to be relied upon as one of two doctors with specialist skills. At the time of writing this letter, proposals to amend the MHA to allow only one doctor to detain a patient were under consideration owing to the Covid-19 pandemic, focusing our concerns into even sharper relief. Dr Gupta's letter states that we attribute the recent 47% rise in detentions primarily to the issues we have raised. Our article does not make this claim and we detail some other likely contributory factors. However, this very significant rise in detentions gives an additional reason to raise the standards of the accreditation process.

We agree with Dr Khwaja's statement that the approval courses can allow for debate around the intricacies of mental health law but assert that this should occur as well as, rather than instead of, ensuring basic competencies.

We of course accept Dr Ballantyne-Watts comment regarding the very low response rate to the questionnaire but want to highlight that the survey was conducted to try to ascertain what degree of standardisation there was between approval courses; we do not base our conclusions upon the questionnaire. We found the lack of engagement from course organisers frustrating and concerning, and this was a driver for writing the article. We plan to survey attendees of the approval courses to shed further light on this issue and perhaps the issue of smartphone usage and expect a much higher response rate.

We know of no other accreditation process in medicine that takes place with the lack of rigour described in this letter and in our article. We accept that the question of requisite knowledge and skills in Section 12-approved doctors is an open one and would again welcome the opportunity to collaborate on performing a study to address the need for more up-to-date research in this field.

